# Mechanisms for stimulating facility quality improvement: A positive deviance study of Tanzania’s Star Rating Assessment

**DOI:** 10.1371/journal.pgph.0006078

**Published:** 2026-03-11

**Authors:** Sanam Roder-DeWan, Anna Gage, Donat Shamba, Heller Rajab, Magreat Somba, Mohamed Mohamed, Mary Ramesh, Talhiya Yahya, Eliudi Eliakimu

**Affiliations:** 1 Department of Community and Family Medicine, Dartmouth Health, Lebanon, New Hampshire, United States of America; 2 The Dartmouth Institute for Health Policy and Clinical Practice, Dartmouth Geisel School of Medicine, Hanover, New Hampshire, United States of America; 3 Health, Nutrition, and Population Global Practice, The World Bank Group, Washington District of Columbia, United States of America; 4 Institute for Health Metrics and Evaluation, University of Washington, Seattle, Washington, United States of America; 5 Health Systems, Impact Evaluation and Policy, Ifakara Health Institute, Dar es Salaam, Tanzania; 6 Department of Disease Control, London School of Hygiene & Tropical Medicine, London, United Kingdom; 7 Department of Quality Assurance, Ministry of Health, Dodoma, Tanzania; 8 East, Central, and Southern Africa Health Community, Arusha, Tanzania; 9 Department of Quality Assurance, Ministry of Health, Dodoma, Tanzania; University of St Andrews, UNITED KINGDOM OF GREAT BRITAIN AND NORTHERN IRELAND

## Abstract

Tanzania launched the nationwide Star Rating Assessment in 2015 and has implemented it as a cornerstone of its national quality improvement program. We use variation in assessment results to explore mechanisms through which facilities improve quality. A mixed-methods positive deviance approach was applied by ranking all primary care facilities included in the Star Rating Assessment (n = 5,595) by their change in score between 2015/16 and 2017/18. The least- and most-improved facilities (n = 27) were selected for qualitative interviews with the highest-ranking provider at the facility on the day of data collection. The dataset was thematically analyzed first as a full-set and then divided by most and least improved to develop a local theory of improvement. Interviews were conducted with 27 facility leaders in 27 primary care facilities. Analysis showed that the SRA helped providers and staff find meaning in their clinical work which served as a core mechanism for improvement. This meaning-making was supported by stronger relationships and connections between health system actors. Connection to leaders came through the creation of a shared national mission to improve quality and an accountability system that allowed respondents to advocate for change. Connections to colleagues came through greater collaboration within facilities and competition between facilities. Connection to patients came through improved knowledge about clinical and interpersonal quality and self-checking against this new knowledge. Inputs to care were important, but not sufficient to drive improvement. Distilling SRA into a replicable improvement model carries with it the risk of losing contextual and programmatic factors which appear to be key to the success of the program in Tanzania. Implementation and ownership by the government of Tanzania was one of these critical elements.

## Introduction

Improving quality of health service delivery has been a priority in Tanzania since the health sector reforms of the 1990s [1,2], throughout the implementation of the Millennium Development Goals [3,4], and into the Sustainable Development Goals era. Despite this longstanding commitment and clear improvements in public health, quality of care continues to be a challenge [5]. Patient visits often lack core components of quality services and outcomes linked closely with the quality of these services miss national targets. Neonatal conditions, for example, are still the leading cause of death and disability in Tanzania, despite an increase in facility birth rates [6].

In response to these challenges, the Tanzanian government has renewed its commitment to providing health services that meet the quality standards necessary to improve outcomes, in part by implementing an ambitious nationwide facility quality assessment and rating system, the Star Rating Assessments (SRA) [7]. Nearly 7000 facilities were given a rating of zero to five stars in 2015 and 2016 during the baseline assessment and the data was used to develop facility quality improvement plans to which providers and leaders were held accountable. Results were remarkable for poor baseline quality - a third of facilities scored zero stars in 2015/2016. A second round of SRA occurred in 2017 and 2018 showing significant improvements in quality; three quarters of facilities improved between the two surveys but with wide variation between the two rounds [8,9]. In a prior study of the contextual factors associated with this variation, we found geographic clustering of higher-performing facilities suggesting that facilities influenced each other. Other determinants of improvement included district administration, facility type (lower-level facilities improved less) and *ineligibility* in a results-based financing program. The last finding suggests that the possibility of becoming eligible was a greater incentive than the program itself [8].

Follow-on studies of SRA data have demonstrated that significant improvement is still needed in facility management and governance, [10–12], patient-provider interaction [13,14], and laboratory services [15]. Nevertheless, the strong political commitment, clear quality metrics, and wide stakeholder engagement in improving services [16] have led to notable progress since the launch of the SRA. The “*Tanzania Demographic and Health Survey and Malaria Indicator Survey 2022”* has shown that the maternal mortality ratio has fallen significantly to 104 deaths per 100,000 live births and that nearly 90% of interviewed women now report that they experienced respectful maternity care [17].

This study complements our prior assessment of contextual factors that influence facility improvement by exploring the underlying facility-level mechanisms through which improvement occurred through the SRA. It exploits the variation in facility quality improvement as measured by the SRA to apply a positive deviance (or best performer) analytic approach with the goal of developing a local theory of facility quality improvement. Elizabeth Bradley and colleagues outlined four steps for using positive deviance methods in the study of health care: (i) identify best performers, (ii) use qualitative methods to generate hypotheses about how these organizations perform well, (iii) test these hypotheses using representative samples, (iv) disseminate the evidence with stakeholders [18]. This paper presents the results of steps one and two. For step one, best and worst performing facilities were defined as those with the largest and smallest documented improvement in scores between the two rounds of Tanzania’s SRA. For step two, the study uses grounded theory methods in the tradition of Corbin and Strauss [19]. This particular grounded theory approach was chosen because it is well-suited to exploring understudied *processes* and is designed to generate novel theory from the data [20]. The study team was intentionally composed of academics, including expert researchers from the Ifakara Health Institute, and policymakers from the Tanzania Ministry of Health (MoH).

## Methods

### Study design and sample

A mixed-methods positive deviance approach was used to answer the research question. First, quantitative data on facility quality improvement was used to subdivide a national sample of facilities into most- and least-improved. Qualitative methods were then applied to explore the mechanisms by which some facilities were better able to improve than others. Definitions and elements of quality health care were based on the High-Quality Health Systems Framework of the Lancet Global Health Commission on High Quality Health System [21].

The data presented here were generated as part of a larger process evaluation of the Star Rating System in Tanzania. The overall evaluation used the SRA results from two rounds of survey data collection (2015/16 and 2017/18) of all primary care facilities in Tanzania (N = 5,595 facilities). A randomly selected representative sample of 208 facilities was selected for the evaluation producing 208 facility surveys, 609 provider surveys, 1,275 client surveys, 1,275 visit observations, and 27 in-depth interviews with facility leaders in 27 facilities. Additional publicly available data sources such as the Tanzania Demographic and Health Survey (2015) were used to complement the primary evaluation data. This paper focuses on the in-depth interviews of 27 facility leaders in a sub-sample of 27 primary care facilities.

The SRA uses facility audits, facility record review and interviews with clinicians and patients to measure health facility performance across four domains: health facility management and staff performance; service charter fulfilment and accountability; safe facilities conducive to health; quality of care. Each domain includes weighted sub-domain scores with overall scores from 0-100%. Stars were assigned based on the domain with the lowest score and as follows: 0–19%, zero stars; 20–39%, one star; 40–59%, two stars; 60–79%, three stars; 80–89%, four stars; 90–100%, five stars [22]. Given that the average change in the overall sample of facilities in the SRA was just under one-star (.98), we defined best performing (most improved) facilities as those that had a two- or three-star change on the SRA between the 2015/16 and 2017/18 rounds of data collection. Worst performing (least improved) facilities were defined as having no documented change or a lower score in round two than in round one. Because we were interested in the two extremes, we did not select from facilities with scores close to the sample average, i.e., 1-star change.

A maximum variation sampling approach was used to purposefully narrow down the overall sample from the 5,595 primary care facilities included in the SRA. In addition to the most and least improved variables, facilities were selected for geographic variation and for facility type (dispensary vs. health center). Given that most qualitative studies achieve saturation between 6 and 30 interviews [23], 27 facilities were initially selected for in-depth interviews with the plan to stop sooner if saturation of ideas (i.e., no new ideas emerging from the data) was achieved, or to expand the sample if saturation was not achieved. The most senior facility staff member available on the day of the visit was approached for the interview.

### Data collection

Participants were recruited and data were collected between July 2, 2019 and August 27, 2019 by a research team trained in research ethics, methods, and implementation of study tools. Qualitative data were collected by two Kiswahili-speaking research staff with masters-level training and extensive experience in qualitative research methods (MR, MS). A semi-structured interview guide designed to answer the study question – *how do primary care facilities improve?* - was developed by the study team in English (see appendix). It was translated into Kiswahili and back-translated to English for accuracy. The instrument was piloted in two facilities after which minor edits were made. After written consent was obtained from respondents, semi-structured interviews in Kiswahili were conducted in private locations on the facility campus or near the health care facility. Private locations for patients were designated by facility staff and included rooms with closed doors or waiting areas if empty. The study staff also gave respondents the option to be interviewed in shaded areas away from the facility buildings. Respondent preference determined the final interview location. Providers were also given the option to be interviewed away from the facility buildings but often preferred to be interviewed in their private offices. Interviews were audio-recorded and interviewers took notes during interviews. In order to protect respondent privacy, no individual names were collected. Interviews were then translated into English and transcribed.

### Analysis

Research team meetings conducted by telephone throughout the data collection period were used to conduct constant comparative data analysis. Emerging ideas, patterns and potential themes were discussed and used to improve the interview tool and to guide future interviews. Interviewers used notes and memos to document and interpret findings and to share data with the larger study team. Study logistics, including the need for translation and the remoteness of facilities, limited the ability to code data during the data collection period.

After data collection, translation and transcription, two researchers (SRD, HR) independently read all interviews line-by-line, proposed, discussed and agreed upon an initial set of open codes, then organized the data using these codes. Open coding led to the emergence of an initial set of themes that together amounted to a *core phenomenon* which was discussed and refined with the larger study team. A core phenomenon is a central concept to which other ideas that help answer the research question are connected. The core phenomenon was then used as the central focus for further axial coding to build out the elements of a grounded theory as described by Corbin and Strauss [19]. These elements are the *contextual conditions* that enabled the core phenomenon to occur, the *consequences* of the core phenomenon, and the *strategies* that respondents took to convert the phenomenon into the consequences. The *causal conditions*, another key element of grounded theory in this particular qualitative approach, were explored in a separate quantitative analysis [8]. Axial coding was conducted in two phases: first looking at the data set in-full; and second looking at the most- and least-improved facilities as distinct sub-sets. Final selective coding, with particular attention to codes which diverged between the most- and least-improved facilities, was necessary to bind the elements together in a model, or theory, of how facilities in this sample improved. Dedoose was used for coding and analysis. (Version 7.5.9, SocioCultural Research Consultants, LLC, Los Angeles, CA).

### Ethical approval

This study was approved by the Ifakara Health Institute Institutional Review Board (IHI/IRB/No: 16–2019) and the Tanzania National Institute for Medical Research (NIMR/HQ/R.8a/Vol.IX/3145). Permission to conduct research in health facilities was obtained from the MoH, The President’s Office - Regional Administration and Local Government (PO-RALG), regional and district authorities in each sampled administrative unit. There were no deviations from the study protocol after approval.

## Results

In-depth interviews were conducted with the highest-ranking staff member available at the facility at the time of the interview in 27 facilities (Table 1). Contemporaneous analysis suggested that saturation of ideas was achieved before 27 interviews were completed, though the full set was collected. More than half of the facilities were dispensaries (n = 15); 12 facilities were health centers. Approximately half of facilities were in the Lake Zone of northern Tanzania and half were in the Southern Highlands. Respondents carried a variety of professional titles and included physicians, nurses, and other cadres. A slight majority (56%) were female. The mean years of work experience was 6.7 years (SD = 3.5). The sample included 12 facilities that were classified as most-improved, and 15 that were least improved (Table 2). The change in score for most improved facilities was 31.4 points higher than the least improved. No potential respondents declined to be interviewed.

**Table 1:** Sample characteristics.

	Most improved (n = 12) *n (%)*	Least improved (n = 15)	Total (n = 27)
	
*Facility-level characteristics*			
Primary care facility type			
Dispensary	6 (50)	9 (60)	15 (56)
Health center	6 (50)	6 (40)	12 (44)
Geographic Zone			
Lake Zone	6 (50)	7 (47)	13 (48)
Southern Highlands	6 (50)	8 (53)	14 (52)
Ownership			
Public	12 (100)	13 (87)	25 (93)
Private	0 (0)	2 (100)	2 (7)
*Respondent-level characteristics*			
Self-identified title			
ANC/PMTCT in-charge	0 (0)	1 (7)	1 (4)
Assistant nursing officer	1 (8)	3 (20)	4 (15)
Clinical officer	3 (25)	4 (27)	7 (26)
Enrolled nurse	1 (8)	0 (0)	1 (4)
Facility in-charge	1 (8)	2 (13)	3 (11)
Matron	0 (0)	1 (7)	1 (4)
Medical attendant	1 (8)	1 (7)	2 (7)
Nurse assistant	0 (0)	1 (7)	1 (4)
Nurse in-charge	1 (8)	1 (7)	2 (7)
RCH in-charge	3 (25)	1 (7)	4 (15)
Registered nurse	1 (8)	0 (0)	1 (4)
Education			
Certificate in Clinical Medicine	3 (25)	3 (20)	7 (26)
Certificate in Nurse Midwifery	1 (8)	1 (7)	2 (7)
Certificate in Nursing	1 (8)	1 (7)	2 (7)
Diploma in Medicine	1 (8)	1 (7)	2 (7)
Diploma in Nursing	5 (42)	7 (47)	12 (44)
Form Four/basic secondary	1 (8)	1 (7)	2 (7)
Gender (female)	7 (58)	9 (60)	16 (59)
	*mean (SD)*		
Years of work experience	7.33 (3.3)	6.21 (3.8)	6.7 (3.5)
Age	40.9 (11.0)	39.7 (10.0)	40.3(10.3)

ANC = Antenatal care, PMTCT = Prevention of Mother To Child Transmission of HIV, RCH = reproductive and child health, SD = standard deviation

**Table 2:** Start Rating scores and ratings.

	Least improved (n = 15)	Most improved (n = 12)
			*mean (SD)*	*mean (SD)*
Star Rating Scores (mean, SD)		
Baseline (2015/16)	45.4 (13.7)	40.8 (14.4)
Endline (2017/18)	49.5	76.3 (20.3)
Change in scores	4.1	35.5
Star Rating (mean, SD)		
Baseline (2015/16)	1.4 (0.63)	0.75 (0.62)
Endline (2017/18)	1.2 (0.86)	2.92 (0.67)
Change in rating	-0.2	2.17

SD = standard deviation; the average change in star rating score for the overall sample was 19; the average change in star rating for the overall sample was.98.

Early in data collection, a core phenomenon began to emerge from interviews: The SRA helped providers and staff find meaning in their clinical work. Participating in the assessment and subsequent improvement efforts drew attention to the significance of their work – significance to the health system leaders, to their co-workers and to communities. The SRA gave frontline workers and managers an opportunity to step out of their service-provision roles to examine what their work means to those it touches. It was a reminder that the quality of care they deliver matters in the clinical moment, of course, but that it also has ripple effects through to the national leaders who sponsored the SRA. Their day-to-day work was connected to a national mission to improve quality. Participating in the program created connections - relationships - between frontline workers and their health system leaders who, through the SRA, demonstrated that the quality of care delivered in that clinical moment is of national importance. Similarly, the quality of their clinical work mattered to colleagues in their own facilities and counterparts in other facilities; their facility star rating score could be a source of pride, of disappointment, or even of envy. Finally, the SRA helped clinicians refocus on why and how the quality of their clinical services impact patients by highlighting the importance of patient experience. It expanded a narrow technical definition of quality healthcare to a broader relationship-based one.

**Fig 1:**
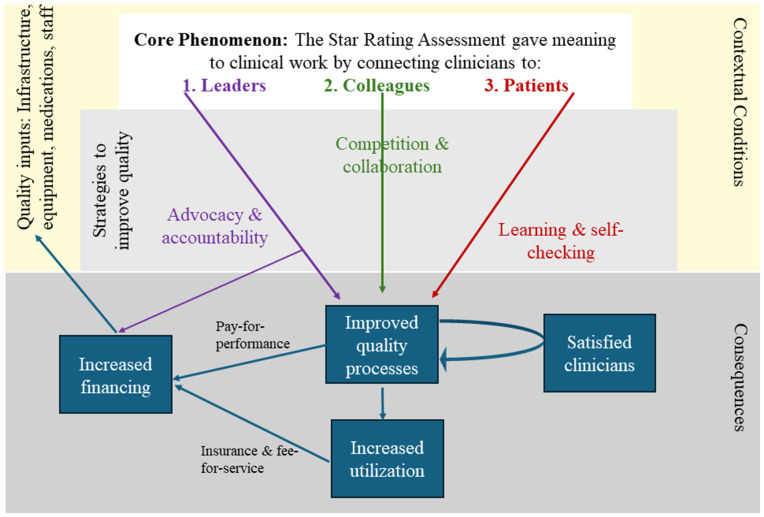
Local theory of improvement.

Using the language of Corbin and Strauss, meaning-making can be considered a quality improvement *mechanism* [19]*.* Health workers used specific *strategies*, to improve quality. These strategies included advocating vis-à-vis decision-makers for needed inputs or changes, collaborating and competing with colleagues and counterparts in other facilities, and self-checking their clinical performance against the SRA standards. The contextual conditions permitting improvement to occur were inputs to the health facility: adequate funding and autonomy to use funds, enough human resources that were competent to deliver quality care, equipment, infrastructure, and supplies. The consequences of these actions, as reported by study informants, were improved quality processes of care, increased utilization of services by catchment populations, and satisfied providers. Data supporting these ideas were largely absent in the worst performing facilities. The local theory of improvement (Fig 1) arose from the full data set and was refined by looking at the codes and ideas that diverged between the most- and least-improved facilities.

### Strategies to improve quality

#### Accountability and advocacy.

Study participants, especially from facilities that improved, spoke about how the SRA brought their facility, its quality, and their individual performance as managers and providers into view for both the MoH and clients. They described “being watched” by the government hierarchy and by communities and welcomed both. Many respondents noted that the SRA was different from other facility quality improvement programs because it was part of the system of accountability, i.e., it was implemented by the government with involvement of officials from all levels. They described the “chain” of accountability – from community to facility, to district, to regional, to national authorities - and noted that SRA helped them improve by strengthening the connection between these levels. Closely related to this stronger chain was the ability to better advocate for facility needs. Respondents described how the SRA gave them a “common language” on quality of care to use when doing this advocacy. Shared expectations on quality of care across the health system levels also facilitated effective advocacy efforts.

“….at times supervisors came by surprise. For example, the ones from the district. So, we were supposed to be prepared all the time. The environment should be clean in and out. So, when they say they come by surprise it is a good thing. It’s fine by me.” *(Clinical officer, most-improved dispensary)*

“So, the management team sits with those questionnaires and development plans and see here we got 0 and here 1, we write aside all that we have failed and see whether this is within our control or not and later we go to the cabinet since they are the ones that authorize and we write to them. So, if there are implementations that are in our control we do them and those that are not, like for example needing a lab technician, then that is not under us, so we write a letter for that. That is how we do it.” *(Clinical officer, most-improved dispensary).*

Staff in facilities that did not improve noted a lack of local ownership over the improvement process.

“[Improvements] are related [to SRA] because there among the people who came and say you have to change, you should do this, do this and that, they are the ones that gave out instructions and those instructions – in case there were weaknesses – we deal with them.” *(Clinical officer, least-improved dispensary).*

“They usually leave a book with suggestions/areas of improvements. My suggestion is that they should go with it and the government should work on them.” *(Facility-in-charge, least improved dispensary).*

#### Competition and collaboration.

Most-improved facilities reported knowing the star rating scores of neighboring facilities and feeling competitive with them, even jealous. They expressed support of the SRA as a fair contest and one they felt they could win if they worked hard enough.

“We had already seen things that got stars in the previous star rating, and how they got money, our fellows got funds the whole year, we were just observers without us getting anything.” *(Facility in-charge, most-improved health center)*

“If one facility does better than the other then the rest will imitate from the one that did better…. there was a time we were called for [SRA] training, so those who did better were questioned on how did they make it, so they explained themselves, so it helps even the others to get ideas from the others.” *(Clinical officer, most-improved dispensary)*

Many described needing to come together as a group to be able to improve performance. Health workers met regularly to discuss the findings of the SRA and their facility responses. These meetings and the shared goal to improve their facility ratings made respondents feel accountable to each other.

“We were all responsible to fight to get stars. All the staff were involved, either its gatekeeper or whoever, it was a big battle.” *(Facility in-charge, most-improved health center)*

Respondents from least-improved facilities did not share similar experiences. Descriptions of competition, benchmarking, and collaboration were not present in the data from least-improved facilities. In fact, least-improved facility respondents sometimes reported not knowing their own SRA scores.

#### Learning and self-checking.

Star rating visits were a learning opportunity for informants. They learned about their weaknesses in delivering care, identified and filled gaps in their knowledge from pre-service education, gained a better understanding of what quality care means, especially related to patient experience, and received updates on clinical guidelines. Knowledge gaps existed because guidelines had changed since respondents had last received training or because they had not received the information during pre-service education. Many respondents described gaining new knowledge that was immediately useful to their clinical practice. They described ownership over the data produced by the SRA (“carrying indicators in my head”) and independence in improving quality.

“When we are in school we are not taught that these information are ours and we use it to improve our services. We were used to taking the information to the district; its theirs.” *(Facility in-charge, most-improved health center).*

“At first, we didn’t have a lot of improvements but after they came it made us want to improve every day using our own efforts by trying to figure out what is missing and what service we are not offering to our clients.” *(Clinical officer, most improved dispensary)*

In contrast, in facilities that did not improve, staff noted challenges with lack of knowledge of the SRA and lack of contact with supervisors outside the facility. They did not describe learning from the SRA or incorporating new ideas of high-quality service-delivery into their day-to-day work.

“To be honest, I didn’t know if there was any other intention [besides quality improvement] because I wasn’t given any.” *(Clinical officer, least improved dispensary)*

“[The District] has reduced the number of visits. They have been crying of budget constraints. It is supposed to be monthly supervision but now a month may pass without any visit…” *(Facility in charge, least improved dispensary)*

### Contextual conditions and consequences

Respondents described the importance of having key inputs in place to improve quality in the context of the SRA. Both most- and least-improved facilities noted the importance of availability and control over funds and human resources as important enablers of improvement, medications and supplies to deliver care. Availability and control of funds was a convergent theme and found in both most- and least-improved facilities. Providers in solo-practice tended to focus on barriers to improvement instead of actions taken to improve quality and inadequate control over quantity and quality of human resources.

“Due to the fact that the budget is coming directly to the facility, we sit and set strategies, and plans, so everything is done on time so there are no things that hinders in improving quality of services.” *(Clinical officer, least-improved health center)*

“We have come a long way, Mama, a very long way. Nowadays the government sends money direct to our facilities. We prepare the budget. In the facility we sit, talk as providers, set our priorities, and present them to the supervision committee of the facility…then we are being sent the money.” *(Facility-in-charge, most-improved health center)*

“Things that help improve, especially the availability of medicines all the time, that has helped a lot in improving our services. And also, our facility has gotten an opportunity to extend buildings, so that is another reason for service improvement, so when people get here they feel nice when they get served on time.” *(Clinical officer, least-improved health center)*

In facilities that improved, respondents recognized that the consequences of their hard work would be felt by patients during their visits because providers were better able to deliver quality care. They also noted that helping patients get better and feel satisfied with the care that they received increased their patient volumes which, through several mechanisms, was directly tied to facility revenue. Separate from this practical incentive to deliver better care, respondents repeatedly described joy in being able to provide quality care.

“I can say even staff have changed. They love their job because they get medical equipment. Also, at the beginning we were very few staff. They added one staff at least now each department has got a provider so it has reduced overworking and everyone loves his/her job because he/she will do the assigned duties and will go to assist a colleague who is overwhelmed with work.” *(Facility-in-charge, most-improved dispensary)*

“If you can attend a patient and return back with positive feedback of the treatment you provided previously it make you happy.” *(Clinical officer, most-improved dispensary)*

## Discussion

The SRA helped providers and staff in primary care facilities in Tanzania find greater meaning in their work through stronger connections to each other, to patients, and to the health system governance structure. With SRA as a platform, informants felt greater accountability to the health system and were able to advocate for support to improve quality. They collaborated with each other and competed with other health facilities over the common goal to deliver high-quality care, and they checked their delivery of health services against newfound knowledge about clinical guidelines and expectations of quality care. A complex interplay of individual, facility-level, and health system factors made SRA a platform for meaning-making and quality improvement.

However, the SRA did not lead to uniform improvements in all facilities in Tanzania. For the SRA to be successful, the infrastructure, equipment, medications, and adequate staffing are important contextual conditions without which participants express dissatisfaction and lack of motivation to improve. The need for these inputs to quality services was a convergent theme and necessary, but not sufficient, to produce quality. Our findings are consistent with the psychology literature on performance in the workplace. Frederick Herzberg and colleagues first described the motivation-hygiene theory in the 1950s and 60s [24,25]. The theory states that the factors which create job satisfaction are different than those that create dissatisfaction and poor performance. An employer may prevent dissatisfaction by addressing contextual or external issues (i.e., the hygiene factors), such as poor working conditions or inadequate compensation, but these improvements alone will not be enough to create satisfied and motivated workers. Factors associated with satisfaction are internal (i.e., the motivator factors), and include a sense of achievement (most frequently), recognition, the work itself, responsibility, and opportunities for advancement and growth [26]. In our study, Star Rating did facilitate action to reduce the burden of challenges through advocacy and accountability channels. However, our core phenomenon linking the Star Rating to improvement was primarily internal. The Star Rating helped participants feel recognized for their work and gave them a sense of achievement. Better understanding how to deliver quality care and feeling more confident in providing good services created satisfaction in the actual work. Participants described feeling self-motivated to check the quality of their work and to improve. Looking at the health care literature specifically, targeting “intrinsic motivation” is consistently shown to produce more significant gains in performance than extrinsic factors alone [27].

The core phenomenon – that the Star Rating helps participants find meaning through connection with others – is also consistent with a rich body of literature on meaning and work. Meaningful work has been shown to increase motivation, satisfaction, and performance and to reduce stress and absenteeism [28]. Meaning for our participants can be understood both sociologically and psychologically; it appears to be shaped by social connections as well as by individual experiences and feelings. Finding greater meaning in work through stronger relationships with co-workers is highlighted in the theoretical literature on meaning in the workplace and, to a lesser extent, has been described empirically. Wrzesniewski and colleagues call this “interpersonal sensemaking”, a process through which workers learn, share, and reinforce values and identity with each other [29]. The SRA also appeared to mobilize connections to other facilities, specifically through benchmarking and competition, a finding which helps explain a prior analysis of SRA scores that showed clustering by geographic area [8].

The SRA is owned and implemented by the Tanzanian government, a fact that participants repeatedly noted. They recognized that government ownership makes the program more sustainable and appeared to weigh its importance more heavily. In a national landscape saturated with international development programs, the SRA stood out as a program that gave health workers visibility within their own accountability system. The program sent a strong signal to participants that quality of care is a priority to the government and helped workers feel that they were part of a broader system and national mission; they were not alone in improving health. The SRA is an example of “governing for quality”, a systems-level centralization of quality improvement as a core mission of ministries of health [21]. The findings that there was “*lack of knowledge about SRA*” has also been documented by German and colleagues in a study that assessed SRA implementation in two councils in Pwani Region in which they noted the importance of providing more support to primary healthcare facilities during SRA implementation and training them on the assessment standards to achieve the full potential of the program [30].

Several limitations should be considered when interpreting these results. First, though we used selection criteria that were informed by Tanzanian health system experts and intended to produce a sample illustrative of health facilities in the country, we are unlikely to have captured the full breadth of experiences. This is especially true for private facilities; though we included private facilities in our study, we suspect that variation within this category is large. Second, we can draw no causal conclusions from the study. The themes we qualitatively describe in this study, however, appear to be associated with improvement via the SRA. Finally, the study was intentionally conducted by a multi-disciplinary team of researchers and policymakers. Though participants were individually consented, interviewed privately, assured that their responses would be anonymized, and reassured that their decision to participate and their responses would not impact their job security, the choice to implement the study in collaboration with the MoH may have created social desirability bias and fear of retribution among participants who were primarily employees of the Government.

## Conclusion

This study identifies meaning-making as a mechanism for improving quality of healthcare in primary care facilities in Tanzania. The SRA helped primary care providers and staff in Tanzania find meaning in their work by linking that work to a national mission to improve quality of care, serving as a platform for intra-facility collaboration and inter-facility competition, and by motivating providers to routinely check their clinical work against technical and interpersonal quality standards. This mechanism, which relies heavily on strengthening relationships between health system actors, is consistent with prior evidence on meaning in the workplace and motivation.

Quality improvement programs may benefit from intentionally targeting meaning-making as a mechanism of action. However, distilling SRA into a replicable improvement model should not be done without considering critical contextual and programmatic factors. Most importantly, implementation and ownership by the government of Tanzania was crucial. Our findings suggest that changing program administration to non-state actors may compromise success and that replication in other countries should consider a similar governance model. Our findings also suggest that similar quality rating programs should include improvement planning with providers and staff and that the work should be conducted in groups to allow for collaboration. Ratings should be shared between facilities to encourage competition. Sharing Star Rating scores publicly, an approach that has been considered by SRA leadership, may further strengthen accountability to communities and is an area of potential future research. Following Elizabeth Bradley’s positive deviance model (i.e., steps 3 and 4), these lessons could be applied to future rounds of the SRA or integrated into other quality improvement programs, prospectively tested, and prepared for multi-stakeholder engagement, dissemination, and adaptation.
